# Medical Consultation Book

**Published:** 1893-10

**Authors:** 


MEDICAL CONSULTATION BOOK. A Pharmacological and Clinical Book of Reference, containing the Therapeutics of a full list of the Officinal and Non-officinal Articles of the Materia Medica, with a consideration of the action of Medicine, including an extensive collection of Favorite Prescriptions from the Most Reliable Authorities of the Medical Profession, and so Classified as to be of Ready Access for Authenticated Treatment of each Disease in its Different Stages and Complications, etc., etc. Designed for the consultation room. By G. P. Hachenberg, M. D., Austin, Texas. Printed at Austin, Texas, by Eugene Von Boeckmann, Printer and Bookbinder. Cloth; 776 pages; price, $7.50.

The general character, aim, and scope of this remarkable book are so fully set forth in the title page, quoted above, that it remains for us to say only, that the author has done his work, as outlined above, well. It is a marvel of laborious, painstaking research and compilation, and is beyond doubt a very excellent work for the purpose of ready reference. And yet it is more than a compilation. The introductory chapters and the "considerations" are original, and do the writer credit. They are written in a style clear and concise, and which would indicate a long familiarity with clinical teaching. The work has met with a courteous reception by the profession, and has been generally commended. It seems to have also met with ready sale, since Dr. Hachenberg informs the JOURNAL that he will at once get out a revised edition, to be printed in Philadelphia. The above book and the TEXAS MEDICAL JOURNAL one year, at the price of the book alone, $7.50. Address this office.

